# PUTTING THE CONTEXT BACK INTO THE DEBATE ON EXTENDED WORKING LIFE

**DOI:** 10.1093/geroni/igz038.3025

**Published:** 2019-11-08

**Authors:** Clary Krekula

**Affiliations:** Karlstad University, Karlstad, Sweden

## Abstract

Policy on extended working life has tended to focus on individuals. The debate has to a great extent described older people as the problem and their current retirement trends as problematic as well as selfish, uninformed, out-dated and a threat to welfare provision and benefits. This depicts the political initiatives as a phenomenon disconnected from social, political and economic trends. This presentation reintroduces the context, by locating the role of policies to extend working lives as forming part of a neoliberal policy agenda. Starting from the understanding that policies are proactive measures which focus on some aspects and play down others, this paper analyses international policies from the EU and OECD and also government policies from 34 European countries. The results draw attention to the narrow and contradictory ways in which the issue is often framed, and how this relegates related new inequalities to the background.

